# Clinical efficacy of ulinastatin in the treatment of unliquefied pyogenic liver abscess complicated by septic shock: A randomized controlled trial

**DOI:** 10.1002/iid3.822

**Published:** 2023-04-17

**Authors:** Mingfeng Guo, Bing Zhou

**Affiliations:** ^1^ Department of ICU The Affiliated Huaian No. 1 People's Hospital of Nanjing Medical University Huai'an Jiangsu P. R. China; ^2^ Department of Hepatobiliary Surgery The Affiliated Huaian No. 1 People's Hospital of Nanjing Medical University Huai'an Jiangsu P. R. China

**Keywords:** clinical effects, pyogenic liver abscess, septic shock, ulinastatin, unliquefied

## Abstract

**Introduction:**

This study determined the therapeutic effect of ulinastatin (UTI) on unliquefied pyogenic liver abscesses complicated by septic shock (UPLA‐SS).

**Methods:**

This was a randomized controlled trial involving patients with UPLA‐SS who underwent treatment at our hospital between March 2018 and March 2022. The patients were randomly divided into control (*n* = 51) and study groups (*n* = 48). Both groups received routine treatment, but the study group received UTI (200,000 units q8h for >3 days). Differences in liver function, inflammatory indices, and effectiveness between the two groups were recorded.

**Results:**

Following treatment, the white blood cell count, and lactate, C‐reactive protein, procalcitonin, tumor necrosis factor‐α, and interleukin‐6 levels were significantly decreased in all patients compared to the admission values (*p* < .05). The study group had a faster decline with respect to the above indices compared to the control group (*p* < .05). The study group length of intensive care unit stay, fever duration, and vasoactive drug maintenance time were all significantly shorter than the control group (*p* < .05). The total bilirubin, alanine aminotransferase, and aspartate aminotransferase levels were significantly lower in the study and control groups after treatment compared to before treatment (*p* < .05); however, the study group had a faster recovery of liver function than the control group (*p* < .05). The overall mortality rate was 14.14% (14/99); 10.41% of the study group patients died and 17.65% of the control group patients died, but there was no statistically significant difference between the two groups (*p* > .05).

**Conclusion:**

UTI combined with conventional treatment significantly controlled the infection symptoms, improved organ function, and shortened the treatment time in patients with UPLA‐SS.

## Graphical abstract

Ulinastatin combined with conventional treatment significantly controlled the infection symptoms, improved organ function, and shortened the treatment time in patients with unliquefied pyogenic liver abscesses complicated by septic shock.

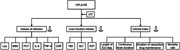

## 1 INTRODUCTION

A liver abscess (LA) is a focal, infectious lesion of the liver caused by microorganisms, such as bacteria, fungi, or amoebas via arterial, venous, biliary tract, and direct dissemination, and is one of the more common acute critical illnesses in the intensive care unit (ICU).^1^, ^2^ Pyogenic LA (PLAs) account for >80% of LA morbidity. The incidence of PLA is higher in Asia compared to Europe and the United States, with an annual incidence of 1.1−5.4/100,000 in China^3^, ^4^ and up to 17.6/100,000 in Taiwan, and 1.0−4.1/100,000 in European countries and America.^5^, ^6^, ^7^ Due to the rapid advances in medical technology, the diagnosis and treatment of PLA have greatly improved, resulting in a decrease in mortality rates from >80% at the beginning of the previous century to <10%.^8^, ^9^ In the most recent decade, the changing diet and aging of the global population, coupled with the emergence of diabetes, malignant tumors, and multidrug resistant highly virulent pathogens, account, at least in part, for the annual increase in incidence and mortality rate of PLAs.^10^, ^11^, ^12^

The direct hematogenous spread of systemic bacteria, such as bacterial endocarditis or periodontal infections, is known to produce PLAs. Significant risk factors for these processes include immunosuppression, liver transplantation, diabetes, biliary procedures, and intra‐abdominal malignancies.^13^, ^14^ PLAs are characterized by severe clinical symptoms, which may include chills, high fevers, pain in the liver area, nausea, vomiting, jaundice, and other related symptoms. Failure to promptly treat a PLA in the early stages may result in progression to infective shock. Some studies have reported that the mortality rate of patients with a bacterial LA combined with infective shock is as high as 20%.^15^ Early percutaneous catheter drainage among individuals with a PLA has been shown to be protective against a persistent fever.^16^ An unliquefied PLA complicated by septic shock (UPLA‐SS) is one of the most common fatal diseases in the ICU because early abscesses in these patients are not liquefied and cannot be treated by puncture and drainage, and surgical interventions to reduce sepsis. Thus, a UPLA‐SS can lead to multiple organ failure, which can be life‐threatening.^15^, ^17^

The pathophysiologic mechanism underlying infectious shock due to an unliquefied PLAs is primarily^18^ an imbalance in the anti‐ and proinflammatory immune system, which in turn induces immune cell apoptosis, microvascular thrombosis, tissue and organ ischemia/perfusion injury, and ultimately leads to the development of shock. In patients with unliquidated bacterial LAs, microorganisms continuously enter the body, and as the cell walls are lysed, large quantities of bacterial lipopolysaccharides (LPSs) are released into the bloodstream. These LPS molecules then form complexes with LPS binding protein and bind to CD14/TLR cell surface receptors, forming LPS complex receptors. This, in turn, activates intracellular phosphorylation reactions, resulting in inflammation and associated symptoms.

It is known that activation of the MyD88‐dependent pathway to induce an inflammatory response is one of the main pathways leading to infectious shock due to a bacterial LA,^19^ in which p38 mitogen‐activated protein kinase (p38MAPK) is^20^ the principal signal transducing enzyme of this pathway. Reports based on septic shock models have confirmed^21^, ^22^ that the use of p38MAPK inhibitors significantly attenuate some inflammatory factors released by endotoxin and inhibit tissue apoptosis by reducing the activation of proapoptotic proteins induced by the release of cytochrome C in hepatocyte outer mitochondrial membrane permeability.

Urinary trypsin inhibitor, also known as ulinastatin (UTI),^23^ is a protease inhibitor present in human urine, blood, and other tissues. UTI inhibits the activity of enzymes that are involved in the production and release of inflammatory mediators, such as neutrophil elastase and trypsin. By inhibiting these enzymes, UTI reduces the level of inflammatory mediators, which mitigates inflammation and tissue damage. In addition to anti‐inflammatory effects, UTI has also been shown to have antioxidant and antiapoptotic properties, which can protect cells from further damage and improve tissue repair. These properties make UTI a promising therapeutic agent for inflammatory and autoimmune diseases.^24^, ^25^, ^26^

It has been shown^27^, ^28^ that UTI can reduce the level of inflammatory mediators, protect mitochondria, scavenge oxygen‐free radicals and antiapoptotic cells in the endotoxic response by reducing the activation of p38MAPK. Studies have shown^29^, ^30^, ^31^ that UTI effectively modulates inflammatory factor release, reduces endotoxic responses, and improves ischemia‐reperfusion injury of the microcirculation in the treatment of infectious diseases, such as severe pneumonia, severe pancreatitis, and sepsis, thereby reducing clinical morbidity and mortality, and is widely used in clinical practice in China, Korea, and Japan.^30^, ^31^, ^32^, ^33^ There are few reports involving UTI in the treatment of unliquefied PLA. This study aimed to retrospectively analyze the effectiveness of UTI in treating UPLA‐SS and provide a foundation for clinical application.

## 2 MATERIALS AND METHODS

### 2.1 General information

This study was a single‐center, randomized, controlled trial (Register number: ChiCTR2200066693). Ninety‐nine patients (58 males and 41 females; average age, 60.18 ± 9.61 years; age range, 33−77 years) with UPLA‐SS who were admitted to the Department of Intensive Care Medicine (Huai'an First Hospital, Nanjing Medical University) from March 2018 to March 2022 were enrolled in the study. The control group was treated with fluid resuscitation, supplemental oxygen, and nutritional support. The circulation was stabilized and vital organ function was maintained. During the initial stage, carbapenems (imipenem/cilastatin [1 g every 8 h] and biapenem [0.3 g every 8 h]) were administered intravenously. Treatment was then adjusted based on the results of pathogenic drug sensitivity tests, as presented in Tables 1 and 2. In addition to the routine treatment received by the control group, the study group was administered 200,000 units of UTI in a sodium chloride solution intravenously every 8 h for >3 days. All patient examinations, tests, and treatments were approved by the patients and their families. The patients signed an informed consent form, and this study was approved by the Ethics Committee of our hospital (KY‐2017‐134‐01).

**Table 1:** Microbial species detected and antibiotics used in the two groups.

Microbial species/Antibiotics	Study group (*n* = 48)	Control group (*n* = 51)	*χ* ^2^	*p* Value
*Klebsiella pneumoniae*	27 (56.25)	30 (58.82)	0.067	.796
*Escherichia coli*	5 (10.42)	7 (13.73)	0.254	.614
*Enterococcus faecalis*	4 (8.33)	3 (5.88)	0.226	.634
*Staphylococcus aureus*	3 (6.25)	3 (5.88)	0.006	.939
Unknown other bacteria	9 (18.75)	8 (15.69)	0.163	.686
Imipenem Cilastatin	42 (87.50)	40 (78.43)	1.430	.232
Biapenem	4 (8.33)	8 (15.69)	1.255	.263
Other	2 (4.17)	3 (5.88)	0.153	.696

**Table 2:** Antibiotic susceptibility patterns of bacterial isolates from the two groups.

Antibiotics	Study group (*n* = 39)			Control group (*n* = 43)		
	*R*	*I*	*S*	*R*	*I*	*S*
Ampicillin	31	6	2	38	4	1
Piperacillin	33	5	1	41	2	0
Cefoperazone sulbactam	18	5	16	15	2	26
Ampicillin sulbactam	35	4	0	39	3	1
Piperacillin tazobactam	17	12	10	29	7	7
Cefazolin	38	1	0	40	3	0
Cefuroxime sodium	36	0	3	38	2	3
Ceftazidime	13	8	18	17	10	16
Cefepime	6	7	26	5	3	35
Aminotransomide	18	2	19	20	3	20
Imipenem	0	2	37	0	0	43
Tobramycin	15	3	21	8	2	33
Amikacin	14	0	25	10	0	33
Gentamicin	15	4	20	8	4	31
Levofloxacin	20	2	17	30	0	13
Cotrimoxazole	14	5	20	9	2	32

Abbreviations: I, intermediate; R, resistant; S, susceptible.

### 2.2 Inclusion and exclusion criteria

The inclusion criteria for an unliquefied PLA were as follows^34^: (1) symptoms of digestive system infection, such as chills, high fever, and pain in the liver region; (2) unliquefied PLA confirmed by ultrasound, computed tomography, magnetic resonance imaging, and other imaging examinations; (3) white blood cell (WBC), C‐reactive protein (CRP), procalcitonin (PCT), and blood cultures confirmed infectious lesions; and (4) in the absence of clear pathogenic evidence, patients were treated with relevant anti‐infection treatment, the clinical symptoms were controlled, and the lesions were reduced and resolved.

The inclusion criteria for infectious shock were based on the 3rd edition of the definition of infectious shock published by the Society of Critical Care Medicine (SCCM)/European Society of Intensive Care Medicine (ESICM),^35^ as follows: patients with severe infection and persistent hypotension despite adequate fluid resuscitation within 48 h after the diagnosis of sepsis requiring maintenance of a mean arterial pressure > 65 mmHg with vasoactive agents, a blood lactate (Lac) level > 2 mmol/L, and evidence of combined acute organ dysfunction, that is, a total sequential organ failure score ≥ 2 from 48 h before infection to 24 h after infection.

**Figure 1:**
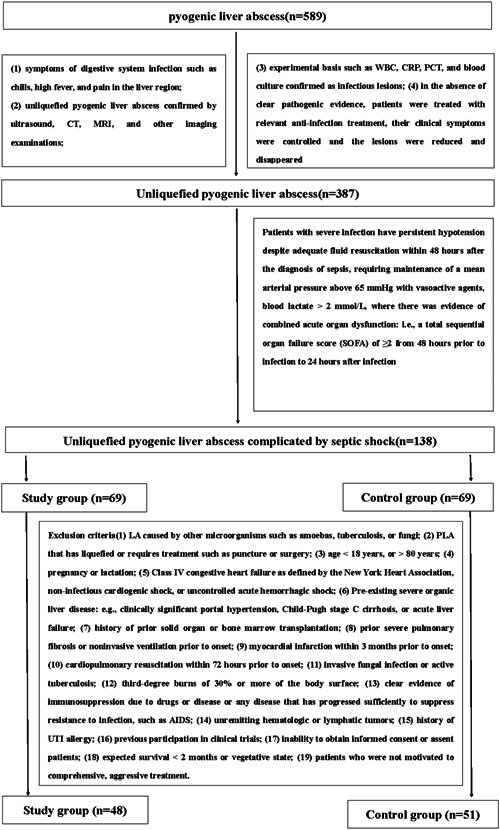
Flowchart of included patients.

The exclusion criteria were as follows: (1) LA caused by other microorganisms, such as amoebae, *Mycobacterium tuberculosis*, or fungi; (2) PLA that has liquefied or requires treatment, such as puncture or surgery; (3) age < 18 years or >80 years; (4) pregnancy or lactation; (5) class IV congestive heart failure as defined by the New York Heart Association, noninfectious cardiogenic shock, or uncontrolled acute hemorrhagic shock; (6) pre‐existing severe organic liver disease, for example, clinically significant portal hypertension, Child‐Pugh stage C cirrhosis, or acute liver failure; (7) history of solid organ or bone marrow transplantation; (8) prior severe pulmonary fibrosis or noninvasive ventilation before onset; (9) myocardial infarction within 3 months before onset; (10) cardiopulmonary resuscitation within 72 h before onset; (11) invasive fungal infection or active tuberculosis; (12) 3rd‐degree burns ≥ 30% of the body surface; (13) clear evidence of immunosuppression due to drugs or disease or any disease that has progressed sufficiently to suppress resistance to infection, such as acquired immune deficiency syndrome; (14) unremitting hematologic or lymphatic tumors; (15) history of UTI allergy; (16) previous participation in clinical trials; (17) inability to obtain informed consent; (18) expected survival < 2 months or a vegetative state; and (19) patients who declined comprehensive, aggressive treatment (Figure 1).

### 2.3 Observation indicators

Infection‐related objective indices were monitored by comparing changes in Lac, PCT, CRP, tumor necrosis factor‐α (TNF‐α), and interleukin‐6 (IL‐6) levels, and the WBC count before and on the 1st, 2nd, and 3rd days after treatment in the two groups. Infection‐related indices were monitored by comparing the duration of ICU stay, duration of fever, and duration of vasoactive drug use in the 2 groups. Liver function‐related indices were monitored by comparing total bilirubin (TBil), alanine transaminase (ALT), and aspartate transaminase (AST) levels, and other index changes before and on the 1st, 2nd, and 3rd days after treatment in the two groups. The clinical outcomes of patients in both groups were followed.

### 2.4 Statistical analysis

The data were analyzed using SPSS 22.0 statistical software, and the count data were described by rate or composition ratio. Normally‐distributed measurement data are described by the mean ± standard deviation, and data with a skewed distribution are expressed by M (range). Comparisons between groups of count data were made using the test or Fisher's exact probability method. Comparisons between groups of measurement data conforming to a normal distribution were made using a *t*‐test, and comparisons between groups with a skewed distribution were made using the rank‐sum test; differences were considered statistically significant at a *p* < .05.

## 3 RESULTS

### 3.1 Analysis of general clinical data of patients in both groups before treatment

The statistical analysis of the baseline and treatment data of both groups did not reveal any significant differences (*p* > .05), indicating comparability between the two groups. The details of the statistical analysis are presented in Tables 1, 2, 3.

**Table 3:** Analysis of general clinical data before treatment in the two groups.

		Study group (*n* = 48)	Control group (*n* = 51)	*t*/*χ* ^2^	*p* Value
Gender (*n*)	Male	31	27	1.381	.240
	Female	17	24		
Age (Year)		61.06 ± 8.20	59.35 ± 10.78	0.884	.379
Abscess diameter (cm)		8.38 ± 3.93	8.4 ± 3.55	0.447	.656
Hepatic abscess site (*n*)	Left lobe of the liver	34	31	1.107	.293
	Right lobe of the liver	14	20		
Number of hepatic abscesses (*n*)	Single	21	20	0.210	.647
	Multiple	27	31		
TBil (µmol/L)		87.37 ± 26.23	84.59 ± 23.21	0.559	.578
ALT (U/L)		272.85 ± 174.02	285.35 ± 194.00	0.337	.737
AST (U/L)		269.52 ± 158.35	262.76 ± 207.83	0.181	.857
Lac (mmol/L)		7.24 ± 1.95	7.13 ± 2.50	0.241	.810
WBC (10^9/L)		16.39 ± 5.96	15.67 ± 6.44	0.580	.563
PCT (ng/mL)	A	41.42 ± 31.31	43.68 ± 38.69	0.318	.751
CRP (mg/mL)		175.02 ± 69.94	168.22 ± 85.34	0.432	.666
TNF‐α (pg/mL)		182.54 ± 79.26	175.45 ± 99.58	0.390	.697
IL‐6 (pg/mL)		251.88 ± 104.35	230.35 ± 141.13	0.858	.393

Abbreviations: ALT, alanine transaminase; AST, aspartate transaminase; CRP, C‐reactive protein; IL‐6, Interleukin‐6; Lac, Lactic acid; PCT, Procalcitonin; TNF‐α, Tumor necrosis factor‐alpha; TBil, Total bilirubin; WBC, White blood cell.

### 3.2 Indices of infection after treatment in the two groups

Post‐treatment, the Lac, CRP, PCT, TNF‐α, and IL‐6 levels, and WBC count in both groups showed a significant reduction compared to pretreatment values (*p* < .05). Moreover, the study group exhibited a significantly faster declining trend in the Lac, PCT, CRP, TNF‐α, and IL‐6 levels, and WBC count compared to the control group, and the difference between the two groups was statistically significant (*p* < .05; Table 4).

**Table 4:** Comparison of objective indicators related to infection after treatment between two groups.

Clinical information		Study group (*n* = 48)	Control group (*n* = 51)	*t*	*P* Value
Lac (mmol/L)	Day 1	6.90 ± 2.27	6.99 ± 2.32	0.195	.846
	Day 2	4.38 ± 1.44	5.09 ± 1.86	2.117	.037
	Day 3	2.01 ± 1.00	2.60 ± 1.04	2.877	.005
WBC (10^9^/L)	Day 1	15.69 ± 3.67	15.25 ± 4.34	0.539	.591
	Day 2	13.49 ± 2.36	14.57 ± 2.81	2.073	.041
	Day 3	12.06 ± 2.31	13.10 ± 2.25	2.267	.026
PCT (ng/mL)	Day 1	37.08 ± 32.43	36.90 ± 24.16	0.032	.975
	Day 2	20.44 ± 15.68	28.05 ± 19.89	2.107	.038
	Day 3	7.51 ± 7.19	14.51 ± 13.60	3.173	.002
CRP (mg/mL)	Day 1	143.35 ± 46.73	148.47 ± 57.26	0.485	.629
	Day 2	112.63 ± 40.10	132.29 ± 53.83	2.052	.043
	Day 3	51.44 ± 27.73	68.25 ± 44.96	2.223	.029
TNF‐α (pg/mL)	Day 1	154.42 ± 69.76	160.35 ± 81.26	0.389	.698
	Day 2	104.96 ± 48.12	128.69 ± 65.88	2.036	.045
	Day 3	64.92 ± 44.32	87.90 ± 59.32	2.173	.032
IL‐6 (pg/mL)	Day 1	203.75 ± 81.20	211.31 ± 100.64	0.410	.683
	Day 2	142.06 ± 63.59	175.35 ± 91.86	2.084	.040
	Day 3	90.81 ± 56.32	129.43 ± 89.01	2.561	.012

Abbreviations: CRP, C‐reactive protein; IL‐6, Interleukin‐6; Lac, Lactic acid; PCT, Procalcitonin; TNF‐α, Tumor necrosis factor‐alpha; TBil, Total bilirubin; WBC, White blood cell.

### 3.3 Indicators of infection after treatment in the two groups

The duration of ICU stay, sustained fever, and vasoactive drug use were significantly shorter in the study group compared to the control group after treatment (*p* < .05; Table 5).

**Table 5:** Comparison of infection‐related indicators between the two groups after treatment.

Clinical information	Study group (*n* = 48)	Control group (*n* = 51)	*t*	*p* Value
Length of ICU stay (h)	150.50 ± 52.33	174.12 ± 57.97	2.123	.036
Continuous fever duration (h)	117.00 ± 51.12	141.18 ± 58.91	2.175	.032
Duration of vasoactive drug maintenance (h)	75.50 ± 34.30	91.76 ± 37.71	2.241	.027

### 3.4 Liver function indices in the two groups after treatment

The TBil, ALT, and AST levels were significantly decreased in both groups after treatment (*p* < .05). Nevertheless, the study group exhibited significantly better recovery of TBil, ALT, and AST levels compared to the control group (*p* < .05; Table 6).

**Table 6:** Recovery of liver function indices after treatment in the two groups.

Clinical information		Study group (n = 48)	Control group(n = 51)	t	*P* value
TBil (µmol/L)	Day 1	72.82 ± 21.45	75.51 ± 20.13	0.644	.521
	Day 2	61.30 ± 19.80	69.80 ± 18.06	2.233	.028
	Day 3	49.07 ± 15.07	57.62 ± 20.16	2.380	.019
ALT (U/L)	Day 1	241.04 ± 108.44	261.06 ± 123.48	0.855	.395
	Day 2	179.31 ± 87.57	219.90 ± 93.56	2.225	.028
	Day 3	102.44 ± 57.77	136.92 ± 64.67	2.792	.006
AST (U/L)	Day 1	230.75 ± 111.83	236.29 ± 121.96	0.235	.814
	Day 2	166.69 ± 93.76	210.65 ± 105.46	2.187	.031
	Day 3	97.27 ± 36.63	140.84 ± 80.05	3.446	.001

Abbreviations: ALT, alanine transaminase; AST, aspartate transaminase; TBil, total bilirubin.

### 3.5 Clinical outcomes

The mortality rate in the study group (10.41%) was lower than the control group (17.65%), although the difference was not statistically significant (*p* = .302).

## 4 DISCUSSION

Infectious diseases affect the entire course of human development, of which there were nearly 50 million new septic patients worldwide in 2017 and 11 million deaths due to sepsis, accounting for 19.7% of global deaths.^36^ An ICU epidemiologic survey in China showed that sepsis affects one‐fifth of ICU patients in mainland China, with a 90‐day morbidity and mortality rate of 35.5%.^37^ The largest etiology of sepsis is septicemia‐induced infectious shock.^37^ Infectious shock, also known as septic shock, is one of the most frequent disorders in the ICU, the onset of which is characterized as acute, critical, and severe, and often combined with insufficiency of one or more organs. If the symptoms of early circulatory failure are not controlled, the morbidity and mortality rates increases sharply.^35^ UPLA‐SS is more worrisome than septic shock alone because the abscess in the inflamed portion of the liver is not liquefied and cannot be treated by puncture or surgery. Thus, the course of sepsis is longer, and the infected lesions continue to release large amounts of endotoxins, oxygen‐free radicals, and other inflammatory mediators, causing patients to develop persistent hypotension and other life‐threatening circulatory disorders.^15^, ^17^, ^18^ Early control of the inflammatory response and protection of organ function are the main measures to correct shock and improve prognosis in such patients.

UTI is a glycoprotein purified from healthy human urine^30^, ^31^, ^32^, ^33^, ^37^ that inhibits the activity of protein hydrolases and consists of 143 amino acids with a relative molecular mass of approximately 67,000. UTI inhibits inflammatory responses and modulate immune function.^30^, ^31^ UTI inhibits the release of inflammatory mediator enzymes, such as neutrophil elastase and hyaluronidase. UTI reduces the permeability of capillaries in acute inflammation, decreases inflammatory exudation, improves local blood circulation, and promotes inflammatory absorption. Moreover, UTI enhances the phagocytosis and adsorption capacity of reticuloendothelial cells, promotes lesion repair, decreases LPS levels by inhibiting p38MAPK, scavenges oxygen‐free radicals, and increases antioxidant enzyme activity, thereby reducing the effects of the SIRS response. Due to a lack of antioxidant defense, oxidative stress is heightened in patients with diabetes. Antioxidants, such vitamin E, assist to repair the antioxidant defense system by reducing the oxidative stress linked to diabetes.^38^

UTI suppresses neutrophil superoxide generation caused by stimuli, such as formylmethionyl‐leucyl‐phenylalanine in a dose‐dependent manner. The reduction of neutrophil superoxide production generated by stimuli, such as formylmethionyl‐leucyl‐phenylalanine, may reflect the fact that UTI reduces the expression of pro‐synuclein‐activated protein kinase, which successfully scavenges endotoxin and protects cellular mitochondria. Patients with sepsis or septic shock who receive UTI have a reduction in all‐cause mortality and other associated outcomes.^39^ A survival benefit trend was reported in clinical studies involving sepsis patients treated with UTI or UTI in combination with thymosin α1.^40^ For endotoxin‐related inflammatory illnesses, such disseminated intravascular coagulation (DIC), acute lung damage, and acute liver injury, UTI has been shown to provide an alluring “rescue” therapy alternative.^41^ UTI has recently been shown to be a key factor in sepsis. It is generally recognized that individuals with sepsis have complicated alterations in their immune system, starting with an early onset of hyper‐inflammatory response and ending with immunologic paralysis. Indeed, no medication has been specifically authorized to treat sepsis in humans. Recent research demonstrated that UTI may protect cells, decrease inflammation, and perhaps improve survival in sepsis and multiple organ dysfunction syndromes.^42^

This finding indicates that UTI has a dual synergistic effect of anti‐inflammation and protection of organ function because UTI inhibits ischemia and reperfusion and enhances the antioxidant defense, thus leading to restoration of the structure and function of hepatocytes. The WBC count, and CRP, PCT, TNF‐α, and IL‐6 levels are the most common clinical indicators to assess infectious diseases.^43^ CRP is elevated in nearly 100% of PLA patients and approximately 74.5%‐91.0% of leukocyte counts are elevated, thus the WBC count and CRP are considered to be simple, effective, and sensitive indicators in patients with PLA to respond to the degree of inflammation and assess the effectiveness of anti‐infective therapy. TNF‐α and IL‐6 are the first cytokines activated and released during the acute infectious period, initiate the SIRS response to sepsis, and are key to the deterioration of patients with septic shock,^43^ while PCT is a diagnostic and prognostic marker of sepsis. Elevation of PCT is positively correlated with the severity of infection, morbidity and mortality, and the sensitivity is also superior to other inflammatory responsive factors.^43^

In this study the trend of improvement in inflammatory monitoring indices, such as the WBC count, and the PCT, CRP, TNF‐α, and IL‐6 levels in the study group were significantly faster than the control group, and the difference between the two groups was statistically significant. After anti‐infection and shock correction, all patients showed a significant reduction in inflammatory markers compared to pretreatment. This finding confirms the effectiveness of UTI in controlling infections and suppressing inflammatory responses with good efficacy, including endotoxin, which is consistent with previous studies on UTI efficacy in treating pulmonary, urinary, and post‐traumatic infections.^30^, ^31^ Patients in the study group showed subjective improvement in inflammation control, as indicated by a reduction in the ICU stay, duration of fever, and maintenance of vasoactive drugs, when compared to the control group, by 16−24 h. This result is consistent with the findings reported by Hu et al.^44^

The liver of patients with an unliquefied bacterial LA, combined with infectious shock, is one of the more heavily involved target organs in these patients, and the production of large amounts of inflammatory mediators in the body during SIRS easily induces apoptosis of hepatocytes, which together with tissue ischemia‐reperfusion injury and microthrombus formation, can easily lead to acute liver injury and even liver failure.^7^, ^15^, ^17^, ^18^ Changes in indices, such as TBil, ALT, and AST, reflect the severity of damage to liver function, and some studies have linked hyperbilirubinemia to eventual death from PLA.^45^ The TBil, ALT, AST, and other liver function indices recovery levels in the current study were considerably higher in the study group than the control group, showing that UTI might lessen hepatocyte damage and apoptosis, thus protecting liver function. The natural products for anti‐inflammatory treatment generally have fewer side effects compared to synthetic medications. Thus, natural products may be better tolerated by some patients and may be a good option for those who are sensitive to or have had negative reactions to synthetic medications. According to reports, administering a high dose of UTI has been shown to significantly delay the onset of decompression sickness (DCS) (*p* = .030) and extend survival time (*p* = .009).^46^ Furthermore, multiple administrations of UTI may yield even greater benefits. Given that steroids are no longer recommended for the treatment of DCS, UTI may be a suitable replacement for glucocorticoids in the treatment of severe cases of DCS.^46^

When a severe infection occurs, tissue hypoperfusion and cellular hypoxia often already exist before the change in routine hemodynamic monitoring indices, and blood Lac levels are already elevated, thus resulting in hyperlactatemia. The elevated blood Lac levels are an early and sensitive biochemical indicator of inadequate tissue perfusion and oxygen delivery, and therefore blood Lac can be one of the important indicators for assessing disease severity and prognosis.^47^ In the results of this study, the decreasing trend in Lac was more pronounced in the study group compared to the control group, indicating that the dual synergistic effect of UTI through anti‐inflammation and protection of organ function significantly improves tissue hypoperfusion and cellular hypoxia in patients with UPLA‐SS, thus improving the prognosis and survival rate of patients. Although there was no statistical difference in the final death rate between the two groups, the mortality rate in the control group was 1.69‐fold greater than the UTI group, thus confirming the preceding findings.

The limitations of the current study were as follows: 1. The specific effect of UTI on antioxidant profiles was not observed. Therefore, further research is expected to address this issue. 2. Studies need to have rigorous study designs, such as double‐blind, randomized, controlled trials to eliminate the effects of other interfering factors on the results. 3. This study was limited to a single center due to resource and time constraints. Further confirmation of our findings with a large multicenter sample is necessary to strengthen the generalizability and external validity.

## 5 CONCLUSION

Combined application of UTI with conventional treatment significantly reduced the inflammatory response, promoted the recovery of organ function, and shortened the treatment time in patients with UPLA‐SS, thus improving the clinical symptoms of patients, which is worth promoting in clinical practice.

## AUTHOR CONTRIBUTIONS

**Mingfeng Guo**: Conceptualization (lead); methodology (equal); data collection and formal analysis(equal); writing—original draft (lead); writing—review and editing (equal). **Bing Zhou**: Conceptualization (supporting); methodology (equal); data collection and formal analysis(equal); writing—original draft (supporting); writing—review and editing (equal).

## CONFLICT OF INTEREST STATEMENT

The authors declare no conflict of interest.

## ETHICS STATEMENT

All the procedures in this study were in accordance with the standards of the Ethics Review Committee of our institution (KY‐2017‐134‐01). Written informed consent from the participants and their families were obtained.

## Data Availability

The data generated or used during the study are available from the corresponding author by request.
